# European Non-Polio Enterovirus Network: Introduction of Hospital-Based Surveillance Network to Understand the True Disease Burden of Non-Polio Enterovirus and Parechovirus Infections in Europe

**DOI:** 10.3390/microorganisms9091827

**Published:** 2021-08-27

**Authors:** Heli Harvala, Kimberley S. M. Benschop, Natasa Berginc, Sofie Midgley, Katja Wolthers, Peter Simmonds, Susan Feeney, Jean-Luc Bailly, Audrey Mirand, Thea K. Fischer, on behalf of the ENPEN Hospital-Based Surveillance Network

**Affiliations:** 1 NHS Blood and Transplant, Microbiology Services, Colindale, London NW9 5BG, UK; 2 Department of Infection, University College London (UCL), London WC1E 6BT, UK; 3 National Institute for Public Health and the Environment, 3721 MA Bilthoven, The Netherlands; kim.benschop@rivm.nl; 4 Laboratory for Public Health Virology, 1000 Ljubljana, Slovenia; natasa.berginc@nlzoh.si; 5 The WHO National Reference Laboratory for Poliovirus, Statens Serum Institute, DK-2300 Copenhagen, Denmark; soi@ssi.dk; 6 Amsterdam University Medical Center, 1105 AZ Amsterdam, The Netherlands; k.c.wolthers@amsterdamumc.nl; 7 Nuffield Department of Medicine, University of Oxford, Oxford OX1 3SY, UK; peter.simmonds@ndm.ox.ac.uk; 8 Regional Virus Laboratory, Royal Victoria Hospital, Belfast BT12 6BA, Northern Ireland, UK; susan.feeney@belfasttrust.hscni.net; 9 CHU Clermont-Ferrand, National Reference Centre for Enteroviruses and Parechoviruses–Associated Laboratory, 63000 Clermont-Ferrand, France; j-luc.bailly@uca.fr (J.-L.B.); amirand@chu-clermontferrand.fr (A.M.); 10 Department of Clinical Research, Nordsjaellands University Hospital, DK-3400 Hilleroed, Denmark; thea.koelsen.fischer@regionh.dk; 11 Department of Public Health and Department of International Health, University of Copenhagen, DK-1353 Copenhagen, Denmark

**Keywords:** non-polio enteroviruses, surveillance, disease burden, emerging

## Abstract

Background. Non-polio enteroviruses (EVs) and human parechoviruses (PeVs) cause a wide range of human infections. Limited data on their true disease burden exist as standardized European-wide surveillance is lacking. Aims. Our aim is to estimate the disease burden of EV and PeV infections in Europe via establishment of standardized surveillance for hand, foot and mouth disease (HFMD) and respiratory and neurological infections caused by these viruses. We will also assess the sensitivity of assays implemented in the network of participating laboratories so that all EV and PeV types are adequately detected. Plan. The European Non-Polio Enterovirus Network (ENPEN) has developed standardized protocols for a prospective, multi-center and cross-sectional hospital-based pilot study. Protocols include guidance for diagnosis, case definition, detection, characterization and reporting of EV and PeV infections associated with HFMD and respiratory and neurological diseases. Over 30 sites from 17 European countries have already registered to this one pilot study, likely to be commenced in 2022. Benefits. This surveillance will allow European-wide comparison of data on EV and PeV infection. These data will also be used to determine the burden of EV and PeV infections, which is needed to guide the further prevention measures and policies.

## 1. Background

Non-polio enteroviruses (EVs) and parechoviruses (PeVs) are known as the main causes of meningitis affecting children and young adults [1,2]. EVs are also a significant cause of other severe neurological conditions including often life-threating encephalitis, acute flaccid myelitis and paralysis. In addition, they cause a wide range of diseases, from mild to severe, such as hand, foot and mouth disease (HFMD), respiratory infections, neonatal sepsis-like infections and myocarditis [1,2,3,4,5,6]. As with other RNA viruses, EVs are characterized by their ability to genetically evolve, which generates potential new variants with unpredictable pathogenicity and epidemic potential [7,8]. PeVs have received limited attention from the scientific community in the past, but continuous reports of PeV circulation all over the world have slowly increased the awareness of their clinical significance [9]. Type 3 PeVs have been most frequently associated with severe disease such as sepsis-like illness and meningitis in neonates and infants [5,6,9,10]. Despite these well-established links, no European data on the true disease burden exist, as standardized European-wide surveillance is lacking [11], disabling early warning and outbreak control. The lack of disease burden data hampers the development and prioritization of targeted antiviral treatment or vaccine development. EV and PeV infections are consequently often poorly managed clinically, leading to likely unnecessary suffering, permanent disability and even mortality among children and young adults in Europe.

In recent years, enterovirus A71 (EV-A71), enterovirus D68 (EV-D68), coxsackievirus A6 (CVA6) and different echovirus (i.e., E30) infections have been frequently reported in Europe [12]. EV-A71 outbreaks have been mainly reported in the Asia-Pacific region; these have been mostly associated with HFMD, but severe neurological complications have also been reported in children <5 years old [4]. The emergence of a new variant, first described in Germany in 2015, led to an alarming upsurge of encephalitis and/or myelitis cases in Spain in 2016 [10,13], Germany, France and the Netherlands [14]. While EV-A71 outbreaks are generally rarely detected in Europe, small HFMD outbreaks linked to CVA6 and characterized by an atypical form of disease have been reported [15,16,17,18,19]. Although CVA6 is now considered as a major pathogen worldwide, the incidence of EV-related HFMD and community-related outbreaks is largely unknown in Europe. Another EV type, EV-D68, has been linked to respiratory infections in Europe and, since 2014, also to neurological complications, namely, acute flaccid paralysis/myelitis (AFM) [20,21,22]. The previous AFM outbreaks in the USA have been mirrored in the European Union (EU) and the European Economic Area (EEA) [20,21,22,23]; EV-D68 cases have been detected in most European countries with knowledge and capability for identifying these infections and AFM cases.

Diagnosing and monitoring EV and PeV infections are complex tasks, especially when many of the 116 EV types are known to circulate simultaneously [24]. A recent large-scale analysis of EVs identified by reference or national surveillance testing in 24 European countries revealed a remarkable total of 66 different types circulating over the 2-year study period [12]. Specimens used for diagnostics depend on clinical manifestations and include cerebrospinal fluid (CSF), stool, respiratory specimens, urine and blood [24]. Molecular methods based on nucleic acid in vitro amplification have become the gold standard for diagnosing EV infections, due to the high sensitivity and specificity and short turnaround times. Sequencing of part of the VP1 capsid protein gene is used for EV type identification [24]. Next-generation sequencing (NGS) methods have also been developed; they are useful to fully characterize circulating EVs and identify possible new variants in surveillance samples [25]. The advantages of NGS still need to be introduced in clinical virology diagnostics, and it may especially be a useful tool for public health surveillance. Virus isolation and other “classical” methodologies including neutralization assays should not be used in primary routine diagnostics due to their known insensitivity and slowness, but the ability to use these methods should be maintained at least in reference laboratories. Despite this, EV diagnostics in many European laboratories still rely on slow and laborious virus isolation together with antigenic characterization of isolates [11,24]. It is also uncertain how widely PeV diagnostics have been adopted in the EU/EEA region.

While studies on the severity of EV and PeV infections and related morbidity are increasingly being published, the data are difficult to compare due to differences between the years studied, clinical cohorts used and how the countries conduct diagnosis and surveillance [12]. Furthermore, testing algorithms and diagnostic techniques, case definition, sample types collected and populations and age groups analyzed have rarely been standardized. In contrast to the existing poliovirus surveillance network, globally coordinated by the World Health Organization (WHO), several different unconnected national surveillance systems are used for monitoring other EV infections. Japan has adopted a sentinel-based EV surveillance system capturing patients with aseptic meningitis, HFMD, herpangina and acute haemorrhagic conjunctivitis [26], whereas surveillance in China focuses primarily on HFMD [16], and in the United States, it is based on the voluntary reporting of EV and PeV typing data [27]. In the EU/EEA region, most countries have established laboratory-based national surveillance systems for EV detections where diagnostic laboratories will send selected samples for further typing at the national reference laboratory—over 5000 samples are typed annually by such systems [11,12]. However, none of this constitutes systematic surveillance, and only some of them collect data on PeV infections.

To address this lack of standardization and coordination of investigations across national boundaries, the European Non-Polio Enterovirus Network (ENPEN) has been recently established. This network of clinical and molecular virologists, clinicians (paediatricians, neurologists and infectious diseases physicians), epidemiologists and public health experts functions in collaboration to develop and share knowledge on diagnostic techniques for EV and PeV detection and characterization, disease presentations and prognosis, virus evolution and pathogenesis [24]. In addition to coordinating collaborative projects and fostering a better awareness of EV- and PeV-related diseases, its overarching aim is to provide data on the burden of these infections in Europe through the establishment of a European-wide surveillance platform for EVs and PeVs which are described in this paper. This is an innovative and challenging task because many European countries have not set up hospital-based surveillance networks previously, and the existing approaches vary between countries. Peer learning will form an important part of this network. Experience from countries where multidisciplinary teams including clinical virologists, paediatricians and public health experts are already working effectively and closely together will be shared to demonstrate how team working can be established and how it can help to achieve set aims.

## 2. Main Aims

In order to estimate the burden of EV and PeV infections in the European region, standardized approaches for surveillance targeting HFMD and respiratory and neurological infections are needed. To achieve this, we have—together with ENPEN members—developed three uniform surveillance protocols for EV and PeV infections. These protocols include guidance for diagnosis, case definition, detection, characterization and reporting of these infections. A second aim is to develop RNA transcript controls and use these to confirm the sensitivity of assays implemented in the network of participating laboratories to detect selected EV and PeV types, as previously described [28]. This is pivotal to harmonize the effectiveness of surveillance introduced into different countries and hospitals.

This work will allow European-wide comparison as well as early detection of newly emerging EV and PeV types and strains. The EV and PeV disease burden will be estimated based on hospitalization rates and rates of admission to intensive care, and by data on mortality. Seroprevalence data will also be collected and used to calculate incidences and inform disease burden estimations. This will, for the first time, provide standardized, large-scale data to allow sound conclusions on the impact of EV and PeV infections in Europe.

## 3. Surveillance Protocols

### 3.1. Development of Hospital-Based Surveillance

The first challenge for the multidisciplinary ENPEN team was to establish a hospital-based surveillance network for EV- and PeV-related HFMD and neurological and respiratory infections. The network was divided into three strands, and each strand began developing a study protocol. The first draft for each study protocol was drafted in the ENPEN establishment meeting in Oxford, UK (10–12 May 2018); these were subsequently circulated to possible members from candidate hospitals, and further communications in order to finalize these protocols between the participating network members were conducted via emails and teleconferences. A total of 36 potential participants from 17 European countries reviewed and agreed on the standardized protocols for the pilot hospital-based surveillance presented here; these countries include Albania, Belgium, Bulgaria, Czech Republic, Croatia, Denmark, France, Germany, Italy, Portugal, Norway, Slovenia, Spain, Sweden, the Netherlands, England and Wales (Table 1).

### 3.2. Study Protocols for Hospital-Based Surveillance

The standardized study protocols for the pilot hospital-based surveillance guide a set of three prospective, multi-center and cross-sectional studies spanning over a 1-year study period, focusing on EVs and PeVs associated with HFMD and respiratory and neurological infections. The main study objective, common to all three study protocols, has been set to collect the most relevant information on EV and PeV infections in a standardized format and to use this information to determine the burden of non-polio EV and PeV infections in Europe.

Cases fulfilling the case definition will be identified from the hospital setting through a routine clinical pathway; they will need to have a laboratory-confirmed EV or PeV diagnosis together with clinical symptoms in keeping with HFMD or respiratory or neurological infection (Table 1). Cases will be enrolled to this study after informed consent has been obtained. Pseudonymized data will be collected using a standardized clinical research form (CRF), consisting of demographic, clinical and laboratory data. Samples will be screened for EV and/or PeV RNA with the PCR method already routinely performed in participating laboratories. Ideally, the method should have been evaluated through an external quality assessment program, such as the RNA transcript controls program described above [28]. EV- and PeV-positive samples will be genotyped either in the participating laboratories or at the corresponding reference laboratory. However, EV and PeV typing is also possible via another ENPEN laboratory if required. The overall incidence of EV and PeV infections will be calculated for participating sites, countries and the whole region; similarly, the specific incidences will also be calculated for certain EV and PeV types (i.e., for the most frequent genotypes and for genotypes related to more severe outcomes). Risk factors of disease severity and complications by EV and PeV subtype will also be assessed. Data on EV and PeV type and sequence will also be collected for molecular epidemiology purposes. Collated scientific data will be published and used by the professional and scientific community as evidence-based information to support the development of guidelines for case management, laboratory diagnostics, surveillance and other public health recommendations in Europe and beyond.

### 3.3. Specific Aspects of the Respiratory Protocol

Although many EVs and PeVs can be detected in respiratory samples, only a few of them have been associated with respiratory disease [1,20,21,22,23]. In recent years, EV-D68 has been identified as a cause of respiratory disease leading to numerous outbreaks worldwide, with several cases developing complications of paralysis [21,22,23]. AFM has been noted as a secondary outcome 3–9 days after a prodromal phase with respiratory symptoms. In this study, we will focus on respiratory infections associated with EV and consider EV-D68 in particular. We will classify respiratory infection into mild symptoms (cough, sore throat, shortness of breath or difficulty in breathing, wheezing, tachypnoea, runny nose, coryza), a clinical diagnosis of pneumonia, bronchiolitis, laryngitis, lower respiratory tract infection or exacerbation of chronic lung disease (i.e., asthma or COPD), or influenza-like illness or severe acute respiratory infection based on currently used definitions at participating sites. In order to fulfil the case definition for EV respiratory infection, EV has to be detected in a respiratory tract specimen. Data on co-infections will be collected as available.

Incidence will be calculated by geographical parameters (site/country/region), by age groups and by gender. Incidence rates will be estimated based on detected cases of the EV subtypes per time period divided by the size of the population at risk. Additional data with regard to EV-D68, an enterovirus associated with acute flaccid myelitis in recent years, will be collected through a questionnaire follow-up at 3 and 6 months post-infection, in order to determine the longer-term consequences of this infection. The epidemiology on circulating EVs associated with respiratory disease in Europe should be studied and compared with data available from the Americas and Asian countries.

### 3.4. Specific Aspects of the Neurological Study

EVs account for the majority of cases of neurological viral infections [1,2,29], whereas PeVs are considered as the second most common cause of viral sepsis-like illness and meningitis in infants. It has been reported that especially children with EV- and PeV-associated neurological infections may have severe neurologic impairment, especially when infections occur in the first year of life or when specific EV types, such as EV-A71 and EV-D68, are involved [10]. The severity and sequelae of these diseases, especially in infants, call for further systematic research of EV and PeV types that are most often involved in neurological infections with the purpose to develop and adopt appropriate case management guidelines and public health measures, including vaccines and antiviral agents.

In the study, incidence will be calculated based on demographic, clinical and virological information collected from a network of study sites. Any patient with suspected neurological infection, meeting the case definition, attending/admitted to a hospital with an estimated catchment area and for whom a clinical sample will be obtained for EV and PeV testing will be included in the study. For laboratory diagnostics, CSF (cerebrospinal fluid) and stool/rectal swabs will be collected [28]. An additional respiratory specimen is desirable for all cases but is obligatory in cases of AFM and AFP to allow for detection of EV-D68. Demographic and clinical information will be collected at first medical examination, and clinical outcomes will be recorded no later than 3 months after the first medical examination of the patient. All data will be collected on standardized forms and in anonymized format. Incidence will be calculated overall (EV or PeV detected from all cases with neurological syndromes), for separate EV and PeV type-associated neurological syndromes (meningitis, encephalitis, meningoencephalitis, sepsis-like syndrome, AFP and AFM) and stratified by demographic (age, gender) and geographical determinants. Additional objectives of the study are (1) to describe risk factors and conditions associated with neurological EV and PeV infections, (2) to identify unfavourable outcomes of these infections, (3) to describe the seasonal and geographical distribution of EVs and PeVs involved in neurological syndromes and (4) to collect data for estimation of the economic burden of neurological EV and PeV infections due to hospitalization and clinical management of severe EV/PeV-associated CNS diseases.

### 3.5. Specific Aspects of the HFMD Protocol

HFMD is a common childhood disease mostly associated with enteroviruses of species A including EV-A71, CVA16 and CVA6 [1]. Most children show self-limited illness typically presenting with fever, skin eruption on the hands and feet and vesicles or ulcerations in the mouth. HFMD, as with most other EV infections, occurs during seasonal epidemics of varying magnitudes during which the different EV types co-circulate and replace each other over time [15,16,17]. In Europe, knowledge on HFMD and on the involved EVs is scarce because surveillance systems focus mainly on neurological EV infections. Moreover, most surveillance systems are for hospitalized patients only, and children with HFMD are usually seen within ambulatory settings and do not require hospital admission. The recent emergence of EV-A71 and CVA6 associated with an upsurge of severe neurological conditions and with (atypical) HFMD outbreaks in Europe justifies closer monitoring of this disease. The HFMD study will focus on children < 16 years old presenting with HFMD (Table 1). HMFD is defined by papular or vesicular rash in at least two of the following sites: palms, soles, buttocks, elbows/knees (external part), oral (or perioral) mucosa. Atypical HFMD will be defined as HFMD associated with papular or vesicular eruption on other sites. Neurological complications of HFMD will be defined as occurrence of clinical signs of central nervous system involvement (WHO guidelines) [30]. An estimation of the incidence will be calculated by participating centers by comparison of numerator data (HFMD-presenting children per month) to denominator data (total number of children attending per month/residing in the hospital catchment area). Appropriate clinical specimens will be collected (any combination of either vesicle swab, throat swab, buccal swab or skin biopsy) according to local routine procedures. Specific attention will be paid to atypical presentations and neurological complications of HFMD and the associated EVs.

### 3.6. Ethical Considerations and Data Collection Tools

Local hospitals or other relevant ethics committees need to be informed of this study, and according to country-specific advice, either verbal or written consent needs to be obtained from each participant. Each participant will be provided with an information leaflet which describes the benefits of their participation. Data will be collected in an anonymized format, and only aggregated data will be shared between countries. Each participating site should be able to demonstrate that they are compliant with the general data protection regulations (GDPR). Discussions are still pending regarding the data collection tool most adapted to the sharing of both clinical and sequence data and suitable to assess the compliance with GDPR rules.

This section may be divided by subheadings. It should provide a concise and precise description of the experimental results, their interpretation and the experimental conclusions that can be drawn.

## 4. Commentary

The ENPEN has, for the first time, created a bridge between different clinical and research groups working on non-polio EVs and PeVs across Europe. These include clinical virologists based at local diagnostic hospitals or reference laboratories, infectious diseases and paediatric specialists serving hospitals, public health professionals responsible for EV infections and scientists with interest and experience on EVs and PeVs. The effectiveness of this approach has already been demonstrated via several publications produced by this group in a short time frame [12,24,25,31,32].

Coordinated hospital-based surveillance has not been previously applied to EVs and PeVs in Europe. It will provide advantages over the usual laboratory-based data collection as it will be able to provide more detailed, purpose-oriented and standardized clinical data on different infections, together with denominator data to be used in burden calculations. It is well established that standardized data are crucial for an adequate evidence-based approach to direct management and surveillance of EV and PeV infections. For instance, as demonstrated last year with the E30 outbreak in Europe [32], no data on the length of hospitalization or associated morbidity were available for an outbreak where over 70% of cases were neurological in nature and most infected individuals were hospitalized. Similarly, although sporadic cases of EV-A71-associated encephalitis have been published from Europe [10,12,13,14], these data were not collected systemically, and hence its clinical impact in Europe remains currently unknown. However, it is important to note that this surveillance will not measure the impact of non-hospitalized EV and PeV infections, and hospitalization criteria may well differ between countries. Further plans for serological and sewage surveillance to capture these milder, or even asymptomatic, EV and PeV infections are being considered by the ENPEN, whereas it will be very difficult to standardize the hospitalization criteria across Europe.

Furthermore, for respiratory EVs, such as EV-D68, a more accurate clinical picture of severe respiratory infections caused by EVs will be obtained from hospital-based surveillance. Based on serological studies, it seems that EV-D68 circulated quite intensively before its detection among severe cases [33,34,35]. It was not until respiratory samples were screened that the virus was identified as a cause of respiratory infection, as usually only stool specimens have been screened for poliovirus and other EVs [25]. In 2014, a coordinated effort for detection of EV-D68 revealed the extensive circulation of the virus in the population in Europe, detecting EV-D68 in patients with respiratory infections [21]. Two years later, a third of European countries were known to routinely screen for this infection. Identification of EV-D68-associated severe neurological infections (i.e., AFM) is currently also challenging as only eight European countries are known to perform acute flaccid paralysis (AFP) surveillance [11]. In general, these infections are underreported, although EV-D68 is known to circulate in Europe. Active case findings led to the identification of 29 AFM cases in Europe in 2016 [22], whereas an EV-D68 task force established in Wales identified over 40 severe infections in Wales alone [36]. As the ENPEN surveillance strands will be linked, data on the progression of respiratory infection to severe neurological infection, such as AFM, can be collected.

Similarly, HFMD surveillance has been established only in one European country thus far [11,17]. Despite that, CVA6 was previously identified as the most commonly detected EV type in Europe, and these infections were associated with HFMD [12]. This surveillance will allow systematic collection of such data for the first time in Europe and will benefit from the French experience in this field. Previous experience shows that systematic and prospective collection of data on EV infection across Europe is required and will enable proper detection of EVs that progress towards severe disease. Extension of the hospital-based surveillance network to cover adults and/or milder infections via general practitioners can be further considered.

## 5. Conclusions

Although we have a good basic understanding of the epidemiology of EV and PeV infections in Europe, we have very little or no data on morbidity and mortality related to these infections. We will investigate the burden of EV and PeV infections in Europe using our recently established ENPEN. By bringing together specialists from different fields including clinical virology, paediatric infectious diseases, academic virology, epidemiology and public health from 17 different European countries, we have created three separate hospital-based pilot surveillance protocols, which are ready to be applied. These data will enhance our understanding and knowledge of these infections. However, this is a pilot surveillance, and hence these protocols will be developed further based on experience gained from this first phase. These data will inform future policies including surveillance establishment and vaccine development and also lead to a better clinical and technical preparedness to detect other possible emerging EVs or PeVs throughout Europe in the future.

## Figures and Tables

**Table 1:** Details of planned surveillance for HFMD and neurological and respiratory infections associated with EV or PeV infection.

	Neurological Infections	Respiratory Infections	HFMD
**Case definition**	Any individual older than 1 year of age: suspected neurological infection with an EV or PeV RNA detected in samples within 3 weeks of symptom onset. Any individual below 1 year of age: suspected neurological infection or sepsis-like illness with an EV or PeV RNA detected in samples within 3 weeks of symptom onset.	Any individual with a respiratory disease considered to have an infectious cause AND a laboratory-confirmed EV or PeV infection.	Any child under 16 years of age presenting with HFMD and EV or PeV RNA has been detected in a clinical specimen.
**Clinical samples**	CSF and stool/rectal swab, preferably also a respiratory tract sample or alternative respiratory tract specimen. In case of AFP/AFM, a respiratory specimen is obligatory to allow EV-68 detection.	A respiratory sample such as a nasopharyngeal swab or aspirate, a throat swab, a combined nose and throat swab, sputum or bronchoalveolar lavage (BAL).	Vesicle, throat or buccal swab or skin biopsy.
**Study population**	All individuals.		
**Study period**	1 year—starting date has not been defined yet due to COVID-19 pandemic (it was initially 1 May 2020).		
**Inclusion criteria**	Any case meeting the case definition, attending or admitted to hospital who has given informed consent and for whom a clinical sample was tested for EV and/or PeV.		
**Exclusion criteria**	Individuals fulfilling the case definition but testing negative for EV and PeV will be excluded but recorded in the study as a denominator.	Individuals with other respiratory virus infections such as influenza or RSV will be excluded but recorded in the study as a denominator.	Non-hospitalized individuals aged over 16 years will be excluded from this study.
**Data collection**	Study site information includes denominator data for population size covered by study site, number of patients overall and with neurological infections by study site and over study period. Laboratory information includes data on methodology used. Patient information including demographics, clinical and virological information. All data will be collected on standardized forms and in anonymized format.	Data collection consists of 3 parts: (1) participation form will be collected at the start of the study, (2) clinical research form will be collected after the initial investigations, and 3 and 6 months after diagnosis and (3) aggregated data on number of individuals attending hospital and tested for EV. All data will be collected on standardized forms and in anonymized format.	Clinical and virological data will be completed by the clinicians and the microbiologist for all children presenting with HFMD and attending a participating pediatric hospital who are tested for EV or PeV RNA. Further denominator data include the population size covered by study site. All data will be collected in an anonymized format, and only aggregated data will be shared between countries.
**Analysis plan**	Data will be processed, analyzed and monitored every month, within 1 week of last submission deadline. Data will be reported 3-monthly, and a more detailed annual report will also be provided to all contributors.		
**Countries with intention to participate**	Albania, Belgium, Bulgaria, Czech Republic, Croatia, Denmark, France, Italy, Norway, Slovenia, Spain, Sweden, the Netherlands and the UK including Wales.	Albania, Bulgaria, Denmark, France, Germany, Italy, Portugal, Slovenia, Spain, Sweden, the Netherlands and the UK including Wales.	Bulgaria, Czech Republic, Denmark, the Netherlands, Spain, Sweden and the UK including Wales.

## Data Availability

Study protocols are available via request to the corresponding author.

## Appendix A

ENPEN hospital-based surveillance network members:

**Name**	**Institute**
Catherine Moore	Public Health Wales Microbiology, University Hospital of Wales, Cardiff, UK
Lubomira Nikolaeva-Glomb	National Center of Infectious and Parasitic Diseases, National Reference Laboratory of Enteroviruses, Sofia, Bulgaria
Lili Pekova	Department of Hygiene, Epidemiology, Microbiology, Parasitology and Infectious Diseases, Trakia University, Stara Zagora, Bulgaria
Maria Pishmisheva	Multiprofile Hospital for Active Treatment Pazardjik, Bulgaria
F. Xavier Lopez Labrador	Center for Public Health Research (FISABIO-Public Health), Generalitat Valenciana, Spain
Javier Díez Domingo	Center for Public Health Research (FISABIO-Public Health), Generalitat Valenciana, Spain
Maria Carberizo	Reference Laboratory for Immunopreventable Viral Diseases, National Centre for Microbiology, Instituto de Salud Carlos III, Madrid, Spain
Cristina Calvo	Hospital Universitario La Paz (Madrid), Spain
Maria Itziar Pocheville Gureceta	Hospital Universitario Cruces (Bilbao), Spain
Maitane Aranzamendi Zaldumbide	Servicio de Microbiologia, Hospital Universitario Cruces (Bilbao), Spain
Juan Valencia-Ramo	Hospital Universitario de Burgos (Burgos), Spain
Nuria Rabella	Hospital Universitario de la Santa Creu i Sant Pau, Universitat Autònoma de Barcelona, Spain
Irena Tabain	Croatian National Institute of Public Health, Croatia
Blaženka Hunjak	Croatian National Institute of Public Health, Croatia
Goran Tesovic	Clinic of Infectious Diseases, Dr. Fran Mihaljevic (CID), Croatia
Elena Pariani	Department of Biomedical sciences for Health, University of Milan, Milan, Lombardy, Italy
Cristina Galli	Department of Biomedical sciences for Health, University of Milan, Milan, Lombardy, Italy
Sandro Binda	Department of Biomedical sciences for Health, University of Milan, Milan, Lombardy, Italy
Laura Pellegrinelli	Department of Biomedical sciences for Health, University of Milan, Milan, Lombardy, Italy
Antonio piralla	Fondazione IRCCS Policlinico San Matteo, Microbiology and Virology Department, Molecular Virology Unit, Pavia, Lombardy, Italy
Federica Giardina	Fondazione IRCCS Policlinico San Matteo, Molecular Virology Unit, Pavia, Lombardy, Italy
Andres Anton	Hospital Universitario Vall d’Hebron, Barcelona Hospital Campus, Catalonia, Spain
Jorgina Vila	Hospital Universitario Vall d’Hebron, Barcelona Hospital Campus, Catalonia, Spain
Robert Dyrdak	Dept of Microbiology, Karolinska Institute and Dept of Clinical Microbiology, Karolinska University Hospital, Stockholm, Sweden
Jan Albert	Dept of Microbiology, Karolinska Institute; Dept of Clinical Microbiology, Karolinska University Hospital, Stockholm, Sweden
Xiaohui Chen Nielsen	The regional department of Clinical Microbiology, Zealand University Hospital, Slagelse, Denmark
Miroslav Petrovec	University of Ljubljana, Medical Faculty, Institute for microbiology and immunology, Slovenia
Mario Poljak	University of Ljubljana, Medical Faculty, Institute for microbiology and immunology, Slovenia
Lieuwe Roorda	Maasstad Ziekenhuis, Rotterdam, the Netherlands
Jaco J Verweij	ElisabethTweesteden Hospital, Tilburg, the Netherlands
Jean-Luc Murk	ElisabethTweesteden Hospital, Tilburg, the Netherlands
Mario Hönemann	Institute for virology, University Hospital of Leipzig, German
Melanie Maier	Institute for virology, University Hospital of Leipzig, German
Svein Arne Nordbo	St Olav Hospital, Trondheim, Norway
Susanne Dudman	Institute of clinical medicine, University of Oslo, and Oslo University Hospital, Medical Faculty, Oslo, Norway
Berg, Are Stuwitz	NIPH Norway
Silvia Bino	Institute of Public Health, Tirana, Albania
Petra Rainetova	National Institute of Public Health, NRL for Enteroviruses, Czech Republic
Ondrej Cinek	National Institute of Public Health, Czech Republic, NRL for Enteroviruses, Czech Republic
Labská Klára	National Institute of Public Health, Czech Republic, NRL for Enteroviruses, Czech Republic
Elke Wollants	KU Leuven, Clinical and Epidemiological Virology, Belgium
Raquel Guiomar	National Institute of Health, Lisbon, Portugal (INSA), Portugal
